# Leaving the Academic Niche–Rhoda Erdmann (1870–1935) and the Democratization of Tissue Culture Research

**DOI:** 10.3389/fcell.2021.801333

**Published:** 2022-02-14

**Authors:** Heiner Fangerau

**Affiliations:** Department of the History, Philosophy and Ethics of Medicine, Medical Faculty, Heinrich Heine University Duesseldorf, Duesseldorf, Germany

**Keywords:** Rhoda Erdmann, tissue culture, regeneration, history, 20th century, academic niche, women in science

## Abstract

In the years after Ross Harrison published his pivotal paper on nerve fiber regeneration in 1907, researchers following his line of research presented tissue culture techniques as an extremely sensitive, difficult, and almost occult methodology. When Philip R. White published a manual on tissue culturing in 1954, he declared that he wanted to disenchant this formerly mystified field of study. With a similar aim Rhoda Erdmann had published a comparable manual more than 30 years before in 1922. Her intention was to offer a book that would make the method “a common property of those who want to do biological research in the future.” When science was about to move from little science to big science, Erdmann tried to democratize tissue culture knowledge. Rhoda Erdmann was in many aspects an extraordinary scholar deviating from the norm. She was one of the few women in the field, working as a low-level assistant at the Robert Koch Institute in Berlin before she took the opportunity to work as a research fellow with Ross Harrison in Yale. She was imprisoned during the First World War on the accusation of being a German spy. After she could return to Germany in 1919, she established a laboratory for experimental cell research in Berlin. In 1929 she was one of the first women to be appointed a professor in Germany. The paper focuses Erdmann’s attempts at distributing practical tissue culturing knowledge. Based on her and other scholars’ research work on nutrient media for cell cultures, and the attempts to optimize these basic tools for different species, this contribution examines the hypothesis that this work constituted an academic niche for underprivileged scientists. The paper analyzes whether Erdmann, due to her extraordinary characteristics, had to use certain niches in the academic world (topics, places, techniques, communities) to pursue her research, and whether her attempts at democratizing her techniques can also be read as an attempt to move out of the niche to gain academic recognition.

## Introduction

Regeneration research of the late 19th century concentrated on regeneration and transplantation of limbs and organs in various species (Morgan, 1901; Korschelt, 1907). Since the 1880s the investigation of isolated, embryonic parts had its place in the methodological arsenal (Oppenheimer, 1971, 1978). Not only full organs but also single cells—such as blood cells, spermatozoa, or egg cells—were isolated and observed in vitro. In addition to spectacular results that drew public attention, such as Alexis Carrel’s cultivation of a chick embryo heart, kept viable and beating for several weeks (Turney, 1995; Landecker, 2007, 68–106), experimentation also began to develop culturing techniques, appropriate culturing media, and the constituents of media, which facilitated growth and development. The underlying idea was that it was possible to keep cells not only alive in culture media but to offer them an environment that allowed the study of growth and development as if they were still in their united cell structure under in vivo conditions. It was in 1907 that Ross Harrison published his influential paper on developing frog nerve fibers, which finally linked regeneration and cellular research. His experiment has ever since been reported as the initiating point in tissue culture research (Maienschein, 1983, 2011). A new academic field—“the cultivation of tissue”—was born, which the Danish biologist Albert Fischer 20 years later described as: “the method which deals with permanent strains of various tissues” (Fischer, 1925, 23).

Nutritive media were a crucial but often hidden element of this research. At first, balanced salt solutions (the famous Ringer solution from 1882) and natural media were used to raise single and/or connected cells. This period was followed by attempts at developing synthetic media (since ca. 1910) which resulted after 1945 in chemically defined, industrially produced media. Today, culture media play a key role in various approaches to the study of cell function, proliferation, and regeneration (Xu et al., 2020).

This paper will focus on the interwar years of tissue culture research as a branch of regeneration studies. In this period, experimental biology—especially research concentrating on regeneration and associated topics—was still an emerging field. In particular, the number of studies focusing on transplantation (connecting extirpated tissue to living tissue) and explantation (surrounding extirpated tissue with non-living substances) increased between 1910 and 1930, as illustrated by a growing number of research publications (Carrel, 1912; Carrel and Burrows, 1912; Erdmann, 1921, 1926), and handbooks (Erdmann, 1922; Strangeways and Thomas, 1924; Fischer, 1925). Looking back in 1940, Eugen Korschelt noted that explantation was a “just established and quite modern working field”, but that its expansion demanded its own section (together with regeneration and transplantation) in his review of the previous 50 years of biological research (Korschelt, 1940, 19f.). Tissue culture’s original domain was the study of growth, development, and cell differentiation. With this starting point, the technique had links to regeneration and explantation research, and effects on various other linked fields such as immunology, cancer studies, and (with limited success) surgery, or reproductive biology and medicine. Nevertheless, retrospectively, “only a small “sect” of researchers embraced early tissue culture as a methodology to investigate the pathogenesis of disease” (Vertrees et al., 2009, 150). One member of this little group was the German Rhoda Erdmann, who was to become one of the first female professors in Germany.

The roles of Ross Harrison and particularly Alexis Carrel in regeneration research and tissue culture have received considerable attention (Bang and Frederick, 1977; Witkowski, 1979; Maienschein, 1983; Turney, 1995; Ambrose, 2019). Brief overviews exist regarding the development of nurturing media (Morton, 1970; Gruber and Jayme, 1994; Vertrees et al., 2009; Yao and Asayama, 2017). For detailed accounts that contextualize the history of tissue culture in the 20th century, far beyond the 1940s, one can turn to Landecker’s book (Landecker, 2007), or, for developments in Great Britain, Wilson’s study (Wilson, 2011). Harrison and Carrel truly became science celebrities during their active years. Rhoda Erdmann’s biography has also been discussed in some detail, though this took until the 1980s, starting with a thoroughly researched doctoral thesis (S. Koch, 1985). Meanwhile her legacy has been saved by historians from sinking into oblivion (Hoppe, 1989, 2012; Schneck, 2000; Jasch, 2017; Vogt, 2018). That said, her life per se is an exciting story that deserves to be told: imprisoned in the United States during the First World War at the beginning of her academic life, she made an academic career in turbulent times, against resistance, to be accused and imprisoned in Germany after the National Socialists came to power.

In my account, I shall focus on Rhoda Erdmann’s role in the development of tissue culture research as a biological discipline. I shall take the hypothesis that this emerging field offered an academic niche that allowed a woman to pursue academic life at a time when most of her colleagues were male. For that purpose, I shall offer a conceptual framework for the idea of tissue culturing as a niche within regeneration research, and explain how far that niche offered opportunities and risks for a woman like Rhoda Erdmann at the beginning of her career. After an overview of her life and work, as well as her self-constitution, I will then argue that her effort to establish tissue culture techniques as a general biological practice, and an academic discipline in Germany, can be seen as an attempt at leaving the niche by democratizing knowledge—a goal she was only partially able to achieve.

## Tissue Culturing as a Niche of Regeneration Research

There are several works on the evolution of knowledge, science as an evolutionary system, or evolutionary epistemology. The basic idea behind these “Darwinian” approaches to describing the development of knowledge is that analogies could be built between the biological evolution of species and the history of scientific concepts. For an overview of the debates about evolutionary epistemology and further literature, one can see the recently published special issue of the Journal for General Philosophy of Science (Gontier and Bradie, 2021). Donald T. Campbell (1974) coined the term “evolutionary epistemology” in an essay about Popper’s theories of conceptual change, arguing that scientific knowledge and its change were the results of variation, trial and error, transmission, selection, and adaptation (Campbell, 1974; Fangerau, 2013). Within this framework, science is not to be understood as a biological sphere. Rather, the ideas of selection, borrowing, and inheriting are transferred from the study of biological species to knowledge and its carriers, and used as if knowledge evolved by the production, selection, borrowing, and inheriting of ideas through scientists and other humans constituting the organizational structure of science, which David Hull has called the demic structure of science (Grantham and Todd, 2000; Hull, 1988).

In a similar analogy the idea of the ecological niche can be applied to science and its organization without equalizing science with an ecological system. In its traditional sense, the term “ecological niche” describes a space with specific ecological site characteristics allowing a species to survive. It is a functional term that does not describe only a habitat but makes the niche a characteristic of a species. The concept has been debated and disputed since its formulation in about 1910. Externalist positions, perceiving environment “as a non-modifiable entity causing evolutionary change in organisms”, stood against constructivist views pointing out that organisms themselves modified their environment, thus creating their own niches (Pocheville, 2015, 558f.). The constructivist idea, especially, has gained momentum, and may serve as a model for the development of knowledge in a scientific context. In any sense, the idea of competition is an important element in the niche concept: niches offer a refugium for species that could not survive the struggle for life under other circumstances, if the dominant species is unable to populate the niche as well. If species compete for the same food, an adaptation of one species to a biotope that the other species cannot access (due to size, climatic maladaptation or other factors) the biotope offers a niche for the otherwise potentially extinct species to survive (Pocheville, 2015). The idea of the “niche” has been translated to academia in evolutionary concepts of the development of science. Here, an “academic niche is an identifiable, circumscribed area of scholarly inquiry that can provide a good match with the individual’s qualifications, interests, and career aspirations”. Thus, besides size, it has “topical, human, methodological and even geographical properties” (Eden, 2008, 734f.).

To some extent, at least in the first half of the 20th century, the field of tissue culture research may be seen as a scientific niche within regeneration research (Engel, 1994, 299). I will illustrate this view by highlighting its topical and methodological niche-features as well as highlighting human and (to a limited extent) geographical aspects that validate the description of this research as a niche.

Regeneration research became a major field of biological research at the end of the 19th century. Previous fascination for limb regeneration in lizards was reformulated into a model for experimental biology, framed by Roux as “developmental mechanics”. Regeneration research seemed to be an ideal field, one that could prove that biology could be understood, at its best, by controlling the influence of specific external factors on growth and development. Or in narrower terms: experimental biologists, following the concept of “developmental mechanics”, perceived the study of regeneration as a model of the fundamental process of the development of living species (Maienschein, 1991; Sunderland, 2010).

Since the 1890s Wilhelm Roux, Leo Loeb, Gustav Born, Ross Harrison, and others, had performed experiments on the explantation and transplantation of cells and tissue to study the survival, development, and regeneration of tissue when removed from its original environment (Oppenheimer, 1971; Witkowski, 1983). The hanging drop method applied by Harrison in his influential experiment had been invented by Robert Koch and was, by that time, a standard method of bacteriology (Landecker, 2007, 39). The concept of “culture”, again, was also well established in bacteriology during the 1880s to describe the multiplication of bacteria in a suitable environment. Robert Koch, for example, used the term “Kultur” in 1876, in a paper describing one of his culturing techniques (R. Koch, 1876). The innovation of Harrison’s and Carrel’s work after 1907 was that they offered ways to observe development over a longer period *in vivo*. As Landecker put it, Harrison’s technique was “able to change the temporal and spatial parameters of observing developing” tissue (Landecker, 2007, 41). Carrell and his assistant, Montrose Burrows, who both coined the term “tissue culture”, transformed the approach with their subsequent research into “a generally applicable tool of experimental biology” (Landecker, 2007, 53).

The “growth or maintenance of explanted tissue or organs” (Gruber and Jayme, 1994, 456) demanded above all a nutritive medium, including a “supporting apparatus or framework” and “growth promoting substances” (Fischer, 1925, 34). These characteristics, together with technical equipment allowing for the continuous replacement of nutritious elements, and the observation of propagating cells, were the unspectacular but essential prerequisites for spectacular results. At first, tissue culturing was above all a technique, a means to an end. But very soon it became a research field in its own right.

**FIGURE 1:**
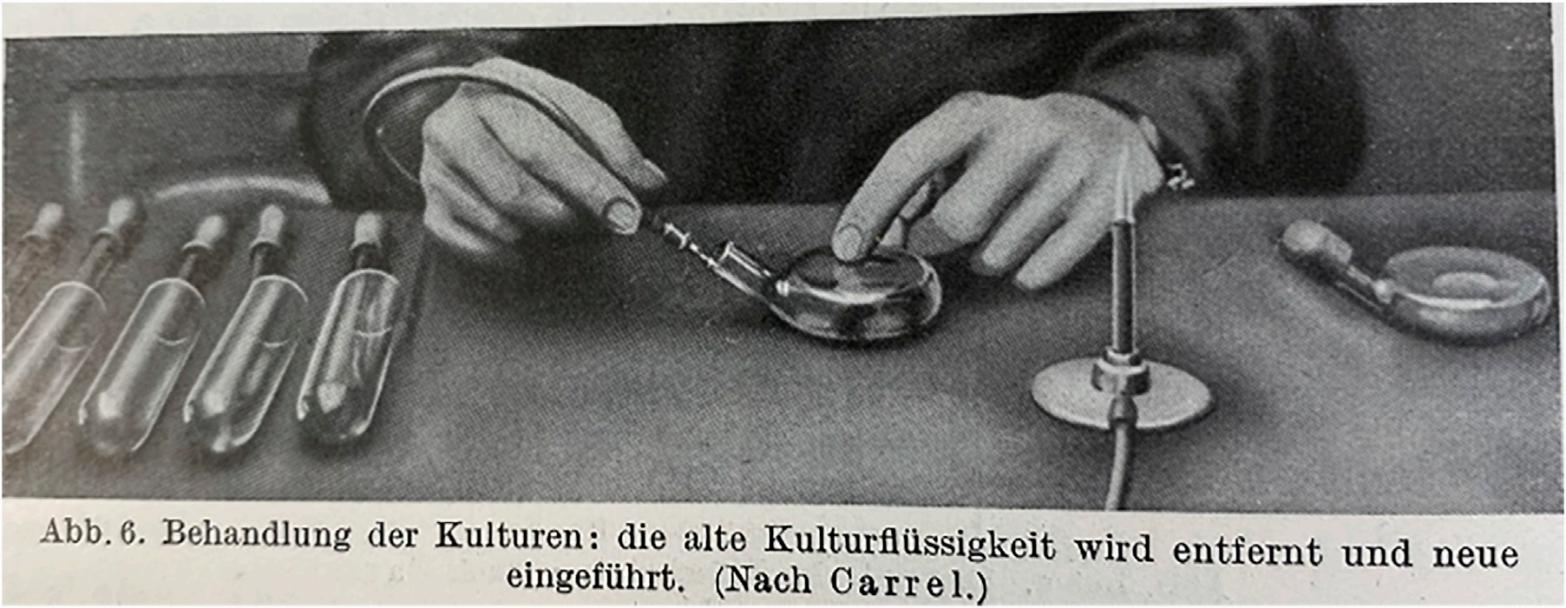
Carrel flasks as displayed in (Bis̀ceglie and Juhász-Schäffer 1928, 34).

Harrison’s method of a hanging drop, in a concavity in the center of a glass slide, was the first standard for observing growth. This method was complemented with hour glasses for larger cultures, Petri dishes, or ring-like object slides, to create chambers for the growing tissue. In 1923 Carrel developed his notorious flask, which allowed a constant flow of fresh nutritional media (Figure 1).

As a medium, clotted blood plasma was first used, because it offered both nutritive elements and a matrix. However, studies very soon experimented with gelatin, hair, cotton threads, or spider webs, as possible frameworks (Fischer, 1925, 27, 44). The materials were tested in various fluids intended to offer nutritive factors. From microbiological research, agar and serum were adopted. Tissue juice originating with the respective cells was also used. The question was no longer about which medium was best for growth and development, but which constituents were the decisive elements. Reducing nutritive media to their core and synthesizing artificial media became the scientific goals. In 1910, Margaret Reed Lewis and her husband Warren Lewis published an article describing the growth of embryonic tissue in artificial media, agar, and bouillon, stating that Margaret Reed had already succeeded with similar experiments in 1908, while working in Berlin at the Institute for Infectious Diseases under Max Hartmann (Rhoda Erdmann was working in the same laboratory at that time) (Lewis and Lewis, 1911b). Not much later, they wanted to take “the next step … to cultivate such tissues in media all the constituents of which” were known (Lewis and Lewis, 1911a, 277). This was the starting point for a series of studies on the role of amino acids, trace elements, and further constituents of nutrient media as the basis of tissue culture. However, it took until the 1950s to prepare standardized media on an industrial level (Gruber and Jayme, 1994), which allowed many more scientists “to work easily with cultured cells” (Yao and Asayama, 2017, 113).

When the German biologist Rhoda Erdmann began her tissue culture research in 1913, she entered a newly emerging field. Ross Harrison (through his formative experiment), Alexis Carrel (through his experimental works on tissue culture after 1910), his co-worker Montrose Burrows, and few others, dominated research in the field, but the number of scientists explicitly engaging in tissue culture techniques as a means and an end was comparably small. Research into tissue culturing did not yet promise immediate success and reputation. Rather, it was perceived as time-consuming and difficult (Gruber and Jayme, 1994, 452; Witkowski, 1979). As Jan Witkowski has shown, Carrel’s “flair for publicity” may have contributed to its image (Witkowski, 1979, 290). Carrel’s announcement that he had succeeded in producing immortal cell lines, and his reports of chicken heart cells still beating in tissue culture, aroused public interest and debates about the limits of science (Turney, 1995; Landecker, 2007). Carrel’s institute, the Rockefeller Institute for Medical Research, fostered prompt publications, which were sometimes perceived by other scientists as reporting unripe results (Corner, 1964, 158). All these factors may have contributed, in the 1920s, to the image of “tissue culturing” as a very promising but “undoubtedly tedious and difficult” (Recent Developments in Tissue Culture, 1924, 72) field of research. Additionally, the lack of immediate medical applications for this research resulted in the (self-)portrait of tissue culturing as a mainly experimental field, which demanded further institutionalization and extension to allow future clinicians to benefit from its findings (Heim, 1928, 80). As The Lancet put it, tissue culture “should be a commonplace in every pathological or biological institute, rather than a field of endeavour for the more adventurous pioneers” (Recent Developments in Tissue Culture, 1924, 72).

Given this background, the subsection of research focusing on culture media, and the practical need for explantation studies, were even more on the margins of biology than tissue culture research itself. Immortal cell lines, as Carrel and others framed it, promised public attention (Landecker, 2007, 68-106). Nutritive media and their components played an important role but belonged to the backstage of regeneration and rejuvenation research.

In the early 1920s, centers of tissue culture research, where media and their role could be studied, were still highly limited. The Rockefeller Institute for Medical Research (Carell, Montrose Burrows), the Carnegie Institute of Washington (Margaret Reed and Warren H. Lewis), the former Laboratory of Harrison and Lewis at Johns Hopkins (Bang and Frederick, 1977), and Yale (Harrison) belong to the United States American pillars. In England Thomas Strangeways established tissue culture research at the Cambridge Research Hospital, founded by him more than a decade before (Wilson, 2005, 2011). In Italy, Guiseppe Levi became one of the most prominent protagonists (Bentivoglio, Vercelli, and Filogamo 2006). However, as the names indicate, research was connected to scientists rather than to places or dedicated laboratories—a situation that persisted well into the second half of the 20th century, and which lead to the foundation of the American Society for Cell Biology as a place for scientific exchange (Brauckmann, 2006). Moreover, some research leading in the direction of tissue culturing, especially before the First World War, took place at de-centralized research institutions such as the Zoological Station in Naples or the Marine Laboratories at Woods Hole. These had been places of international networking and international exchange, which offered as extra-university, sometimes private institutions, special opportunities for researchers who were underrepresented or underprivileged in academia at that time. Examples are the abovementioned Margaret Reed-Lewis (who conducted studies at Woods Hole) and her mentee, Mary Jane Hogue, who benefitted in her research (which included tissue culturing) from stays in both Naples and Woods Hole (Zottoli and Seyfarth, 2015, 143-147, 152f.).

Not only tissue culturing but also zoology as a whole seem to have been characterized, at the beginning of the 20th century, by features that made it easier for women to work in these fields rather than in other sciences. In a review of more than 500 female biographies, Margaret Rossiter (Rossiter, 1974) showed that zoology and botany were the most popular sciences among female United States scientists before 1920 (18.3 and 18.1% working in these disciplines). She mentioned sexual discrimination as one of the potential barriers that women faced when entering science. In the United States, the existence of women’s colleges such as Bryn Mawr, or funding opportunities such as the Naples Table Association for Promoting Scientific Research by Women, which funded research trips to the Zoological Station in Naples (Sloan, 1978), may have contributed to reducing discrimination, at least in the minds of male scientists teaching there. Thomas H. Morgan or Jacques Loeb, for example, worked at Bryn Mawr at the beginning of the 1890s, and kept on promoting female scientists later in their careers (Sunderland, 2010, 334).

One German scientist who entered the niche of “tissue culture” in the United States in 1913 *via* research on Protozoa was the biologist Rhoda Erdmann, who received a Theresa Seessel Research Fellowship at Yale in 1913 (S. Koch, 1985, 16).

## Rhoda Erdmann

Rhoda Erdmann was born in 1870 in Hersfeld, Hessia (on Erdmann’s spectacular biography, see Caffier, 1936; Hoppe, 1989, 2012; Jasch, 2017; Koch, 1985; Schneck, 2000; Wasserman, 2016; Niedobitek, Niedobitek, and Sauerteig 2017, 67–127, 186-209; Vogt, 2018). After her school education, she worked for nine years as a teacher—at that time almost the only academic option for women in Germany (Albisetti, 1989). In 1903 she began to study sciences in Berlin, later studying in Zurich, Marburg, and Munich. In 1908 she was promoted to Dr. phil. by the biologist Richard Hertwig in Munich. Richard B. Goldschmidt had been her supervisor. For her dissertation she had performed cytological studies on sea urchin eggs. In 1906 and 1908, she was able to carry out research at the Zoological Station of Naples, at that time one of the hot spots of biological research. From 1908 until 1912, she worked in the position of an unskilled assistant at the Institute for Infectious Diseases (later Robert-Koch-Institute) in Berlin, where Max Hartmann — also a doctoral student of Richard Hertwig — held a professorship. Her first application to habilitate was rejected by the Prussian Ministry of Culture (Schneck, 2000, 174).

**FIGURE 2:**
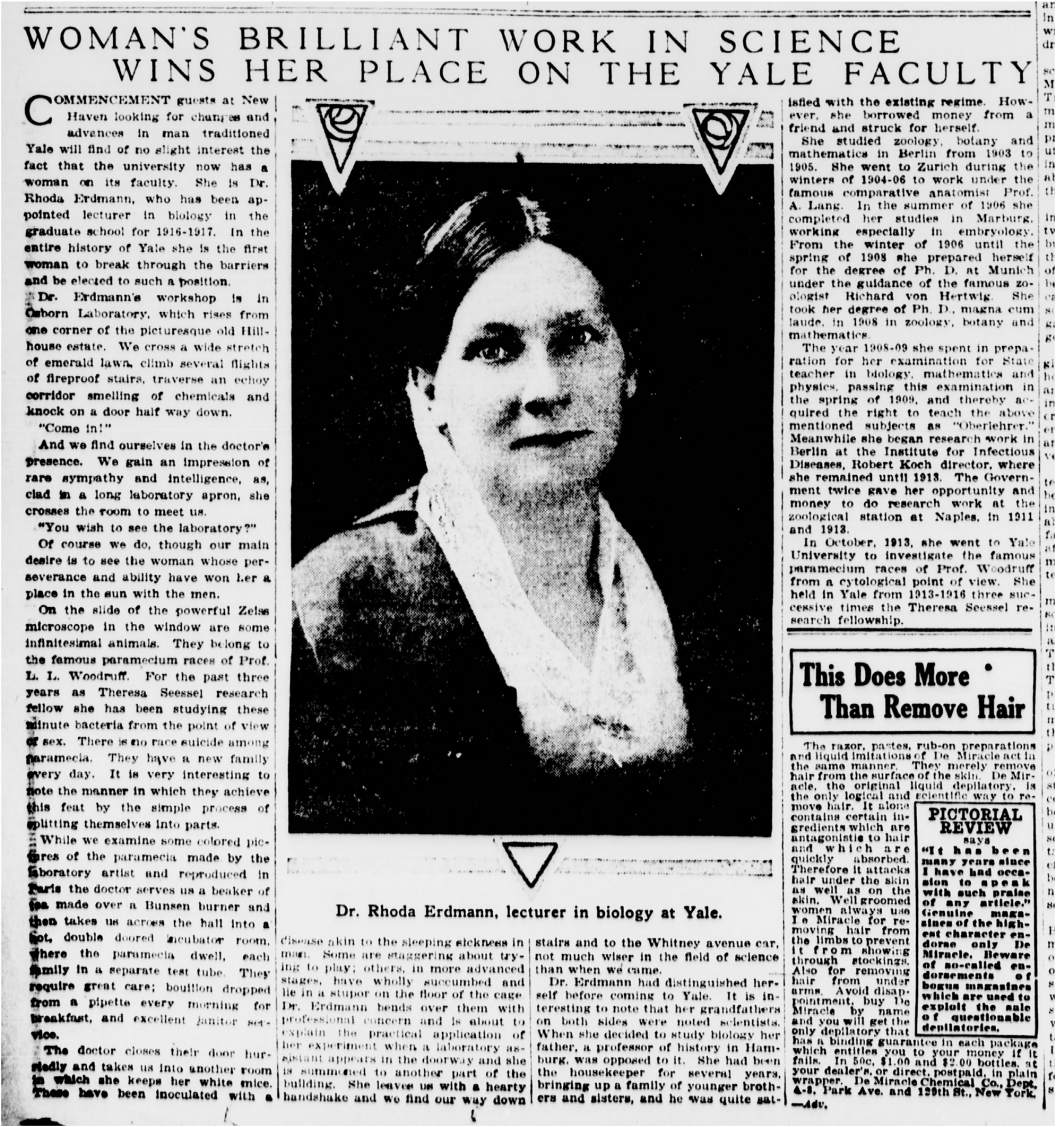
Article about Erdmann’s appointment and work at Yale. The Sun (New York [N.Y.]), 11 June 1916, page 6 (https://chroniclingamerica.loc.gov/lccn/sn83030272/1916-06-11/ed-1/seq-26/ accessed 27.11.2021).

In 1913 she received the abovementioned scholarship, which allowed her to work with Ross Harrison in Yale. Harrison introduced her to the newly established tissue culture techniques. With a scholarship from the Naples Table Association for Promoting Laboratory Research by Women, she was able to go on a short research trip to the Zoological Station in Naples in July/August 1913, from which she returned to the United States (Scientific Notes and News, 1913, 748). On her way back home to Germany in 1914, the First World War broke out and she returned to the United States. Harrison organized a lecturer position for her. The New York Sun published a short very sympathetic report about her being the first “woman to break through the barriers and be elected to such a position” at Yale (Figure 2).

Additionally, she became an associate at the Rockefeller Institute in Princeton in 1916. In the meantime, she tried to return to Germany, hoping for a habilitation and her own department for cell research at the Kaiser-Wilhelm-Institute of Biology. However, her habilitation application to the Ministry of Culture was refused again and her own department did not materialize either (Schneck, 2000, 175).

When the United States joined the First World War in 1917, her prospects clouded. She and her fellow scientist and former supervisor, Richard Goldschmidt, who had also been trapped in Yale when the War started, faced anti-German resentment. They were accused of being German spies and Erdmann was suspected of preparing biological warfare. She was working on immunization by infecting chicken with cyanophilia and the authorities accused her of having imported the pathogen against the law. She was forced to kill her chicken but seems to have kept a jar of cyanophilia, which was discovered (Wasserman, 2016, 15). As a result, she and Goldschmidt were arrested. Media reported the arrest, one with “the gendered headline ‘Fear Woman Scientist’” (Wasserman, 2016, 17).

Goldschmidt was sent to a prison camp for Germans at Oglethorpe. Because this place lacked barracks for women, Erdmann was kept in a house in Manhattan, in one room together with six other Germans under extremely poor conditions (Wasserman, 2016, 32-33). When the War ended she immediately returned to Germany — suffering from a skin infection as “my last souvenir of the prison”, as she wrote to Harrison from aboard the ship (Wasserman 2016, 34). The experience of being ripped from her research, her honor and her freedom cast a long-lasting shadow over her life.

Back in Germany she tried hard to find a job. According to her memoirs, she wrote 59 unsuccessful applications (Erdmann, 1929, 52). Even Goldschmidt, as one of the directors of the Kaiser Wilhelm Institute of Biology, could not ensure a position for her. Finally, the pathologist Johannes Orth created a workplace for her at his Institute for Cancer Research at the Charité in Berlin, which she used to establish a Department for Cell Research at the Institute. New laws finally allowed her to habilitate in 1920. Her inauguration speech was programmatically dedicated to the “importance of tissue culturing for biological research” (S. Koch, 1985, 32-36; Schneck, 2000, 176; Erdmann, 1920). In summer 1924, she was appointed professor as one of the first female scientists in Germany. In 1925, she contributed to the establishment of tissue culturing as an academic discipline by founding the “Archiv für experimentelle Zellforschung — besonders Gewebezüchtung (Explantation)” (Archive for experimental cell research — especially tissue culturing (explantation)). In subsequent years, she tried to establish her department as an institute with its own budget. However, she had to wait until 1930, when her efforts ultimately bore fruit and the department was transformed into a University Institute for Experimental Cell Research (Jasch, 2017; S. Koch, 1985).

Here a real success story could end; but Erdmann had to face the next setback for herself and her discipline when the National Socialists came to power in 1933. She was imprisoned again in 1933, on the accusation of being probably Jewish or socialist, and again, when these allegations proved wrong, on the accusation of having supported Jewish scientists. She was dismissed from the university and reinstalled again. In 1934 she was retired and her Institute was closed (Schneck, 2000; Jasch, 2017). She died on August 23rd, 1935 in Berlin.

## Democratizing Methods as a Means

Erdmann belonged to the first generation of German women who could pursue an academic career. In a way, she served as a role model when she contributed a chapter about her scientific career to a book on “Leading Women of Europe” (Erdmann, 1929). Written in 1926. She explains in detail how difficult it was for women in general, and for her specifically, to compete against male scientists within the existing system. She compares science to a syncytium, in which many cells did the same work to the effect that a minimal advantage could lead to a scientific discovery being attributed to one scientist, although many others had had the same idea. Against this background, women were in her view often eclipsed by men. Women were assigned routine duties such as counting cells, teaching, or supervising students, which prevented them from doing their own research. If women prevailed under these circumstances, they had to face passive resistance from their male colleagues, which she compared to a “herd-reaction” in the sense that men only supported men as their kind. Thus, female scientists ended in isolation without the chance of networking and exchanging ideas. Altogether, the ability of female scientists to execute research was, according to her report, systematically restricted. As a consequence, women had to fight for research spaces, which, and this she considers remarkable, were first given in zoology and botany (Erdmann, 1929, 35–40). She ends her autobiographical report with the statement that women could not use their productive powers because scientific posts produced by men were only given to men and, if a woman wanted to have a “right to exist”, she needed to establish a new discipline of her own (Erdmann, 1929, 54), as she had done.

Although she does not use the word “niche”, her whole report can be read as an account of the difficulties of finding an academic niche and expanding it into a major research field. She might have considered the practice of applying tissue culture techniques and doing research on media as a niche with a dead end, if it was reduced to preparing media. A first small step out of this limited niche into the light of science might have been, for her, a small publication on “A New Culture Medium for Protozoa” (Erdmann, 1914). It was not her first publication but the first explicitly addressing media as a research topic. She had started her scientific works with studies on protozoology and immune biology. Here she became acquainted with the methods of preparing culture media which she could use after 1914 for her works on culturing tissue. She successfully connected culturing tissue and immunology when she was able to show that the pathogen of avian influenza could be attenuated with the help of cell culture transfers. Her research after 1920 encompassed, among other works, the culturing of “immortal” lines from embryonal mesenchymal guinea pig cells, improving the culture of epithelial cells, and work with blood cells in culture. Last but not least her links with the cancer clinic made her focus on culturing cancer cells, and investigating their growth and behavior before and after transplantation (S. Koch, 1985, 50-83; Caffier, 1936).

Her “ergography” (meaning her works’ thematic profile over time) reflects her development, and the fact that she managed to move from the niche of culturing techniques and media to the larger field of tissue culture research, and its associated problems. Her biography shows that she had to carve out her academic standing for herself by hard work. In harsh words she complained in her autobiographical sketch about sexism, male networks, and competition (Erdmann, 1929). To help women actively to create networks, she co-founded the Verband Deutscher Hochschuldozentinnen (Association of German Female Professors) in 1925 (Lohschelder, 1994, 191).

It is true that she found a way into science in Berlin and at Yale by inhabiting the niche of media preparation and culturing. But when she strove to move to more prominent research fields, her struggles and competition inside and outside the niche must have made her bitter and sometimes difficult for her peers. In an episode about her trying to get an automobile in the United States to allow her to work in the laboratory on Sundays, for example, Simon Flexner wearily stated: “I know all about Dr. Erdmann’s troubles. I fear that she demands more than we can give her.”^1^

Simultaneously, she had to cope with an implicit and explicit anti-feminist environment, nurtured even by her friends (see also an episode with one of her assistants described in Satzinger, 2004, 118–121). Harrison, for example, stated that she had “certain unfortunate external traits of character which at times antagonize people,” and Goldschmidt remembered that she had impressed the Americans as an “aggressive spinster type” (Wasserman, 2016, 16). Theobald Smith bemoaned her “streak of intense personal ambition” (S. Koch, 1985, 99). Even her obituarist, her pupil Paul Caffier noted her sometimes difficult character that made her “grim enemies”. That said, Caffier did not shy away from gender stereotypes, stating that Erdmann made these enemies because of her “masculine nature inclined to fight and dispute”. Simultaneously, he noted “she was personally sensitive, a trait that probably stemmed from her womanhood, and not without a healthy ambition … ” (Caffier, 1936, 136).

Pushed back, she did not shy away from conflict. She was self-confident and saw herself as one of the leading scientists in her field. When the Kaiser-Wilhelm-Institute of Biology invited the Danish biologist Albert Fischer (Astrup, 1957) in 1926 to create a guest department for tissue culture, she wrote a ferocious letter asking for an explanation. She explained that everyone knew that she was the one who had introduced tissue culture techniques to Germany and the surrounding states, that she had learned more than Fischer from more important teachers, and had been an associate at the Rockefeller Institute, whereas Fischer had never been more than an assistant. She ended with the statement that she had more enemies than she knew: “People just always try to push a productive woman against the wall. It will be like that forever and will remain like that forever. But I did not assume that a body like the Kaiser Wilhelm Society would stand out so little from what the average person does”.^2^

Additionally, from the beginning of her career she was fighting for resources. Resources were as essential for successful experiments with tissue culturing as for any other field. Lewis Rubin, in his analysis of Leo Loeb’s (often disputed) role in the development of tissue culture, noted that it was a lack of resources in the end that made Loeb shy away from further studies after 1903. He was urged to move to transplantation experiments, which he considered easier to conduct and, when he returned to tissue culture in 1911, Harrison and Carrel had taken over the field in credit and reputation (Rubin and Lewis, 1977, 44–45; see also Witkowski, 1983). At the same time Rhoda Erdmann, like other colleagues, was convinced that tissue culture should be considered as one of the basic methods of biology. On the one hand the method, according to her views, produced evidence for biological knowledge; on the other hand, it saved in the end animal material otherwise needed for biological experimentation (Erdmann, 1920, 1329). She had experienced (like Leo Loeb) that a lack of resources hindered the proliferation of her specialty. In her autobiography and on other occasions, she complained about the lack of resources and support. Especially at the beginning of her scientific career, she had experienced financial problems. When she lost a law case against the publisher Teubner (she had promised a handbook on biology for schools which she did not finish appropriately), she could not pay back the advance payment credited to her.^3^ She was constantly forced to collect funding for her research and, even in later years, she used her limited personal funds to equip her laboratory (S. Koch 1985, 32ff., 38; Niedobitek, Niedobitek, and Sauerteig 2017, 127).

To improve her academic standing and the standing of her research focus, she thought to wrest the tissue culture technology from the hands of prominent experts by the publication of a guidebook on the detailed steps of its practice. She wanted to make the technique freely available for a wider audience. Her practice book was not the first on tissue culturing and not the last, but it was the first to offer explicit exercises. In 1914, Eugenio Centanni had already published a monograph in Italian (Centanni, 1914). Erdmann’s book of 1922 was followed by one from Thomas Strangeways in 1924 in English (Strangeways and Thomas, 1924, not quoting her), another by Albert Fischer in 1925 — originally his Copenhagen dissertation and translated into German by Fritz Demuth (Fischer and Demuth, 1927) — and one by Vincenzo Bis̀ceglie and Alexander Juhász-Schäffer in 1928 (Bis̀ceglie and Juhász-Schäffer, 1928). However, Erdmann’s book paved the way for her next endeavor, the establishment of the “Archiv für experimentelle Zellforschung—besonders Gewebezüchtung”, with the help of which she wanted to create a “center” for the “so far scattered works” on experimental cell research in order to strengthen this “young, but strong branch on the tree of developmental mechanics” (Erdmann, 1925, Preface). To Simon Flexner, who was “by no means convinced that a special journal is called for at the moment for that subject”^4^ she wrote that with the journal she intended to make American works available in Europe and that she wanted to offer a place for works from all over Europe concentrating on tissue culture.^5^

**FIGURE 3:**
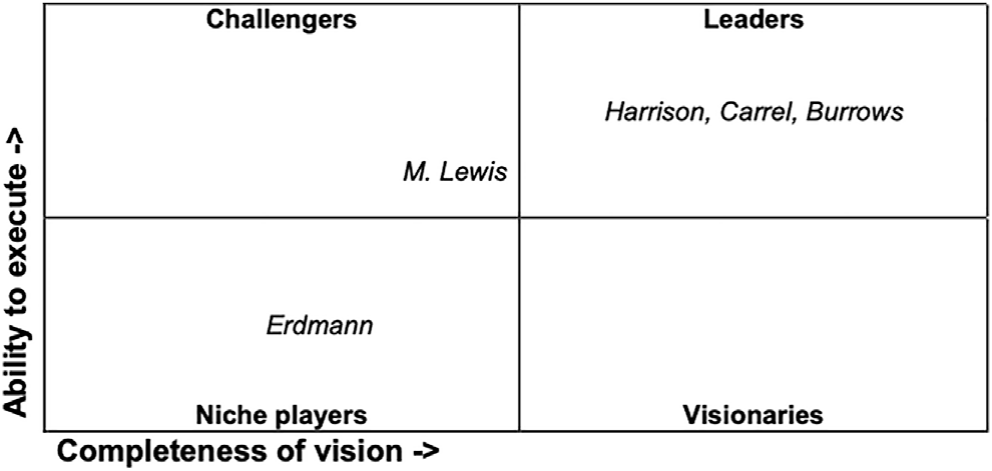
Magic Quadrant Tissue Culture, state around 1920.

A modern model matrix may be applied to illustrate her strategy, on the basis of her struggles and her self-understanding (Figure 3). The business analyst Gartner developed a matrix to illustrate positions and expectations of vendors. The matrix called “Magic Quadrant” has two axes. The first displays a vendor’s “ability to execute”, summarizing “factors such as the vendor’s financial viability, market responsiveness, product development, sales channels and customer base.” The second, called “completeness of vision”, “reflects the vendor’s innovation, whether the vendor drives or follows the market, and if the vendor’s view of how the market will develop matches Gartner’s perspective” (Lehman, 2008). Vendors are positioned in one of four quadrants — named “leaders”, with a high ability to execute and a vision for the future, “challengers”, who have the ability to execute but lack vision, “visionaries”, who are innovative and have a future vision of their field, but lack resources, and “niche players”, who may do well in one segment but lack ability to execute and vision to outperform others.

Applying this admittedly anachronistic and simplistic model to Erdmann can illustrate her strategy. She started her career in the United States as a niche player, with a low ability to execute due to the lack of academic freedom and institutional capacities. This does not mean that the niche is of a lesser quality than the leading field. The niche rather does not offer the same academic visibility and the associated reputation attributed to the assumed leaders of a field and their challengers (on the attribution and staging of recognition and reputation in science see the overview Hansson, Halling, and Fangerau, 2019). Coming back to Germany with new methods and technologies at hand, she was a visionary lacking the ability to execute. The lack of funding and the lack of an institute to direct and teach her own work made her desperate, but she was able to find a way to become, ultimately, a leader in the field. One of her methods, besides competing, was democratizing the methods of tissue culturing.

Tissue culture was perceived and presented, especially by Alexis Carell, as a mystic science located somewhere between witchcraft, alchemy, and cooking, which could only be performed by highly qualified experts having at their disposal enough resources and equipment (Witkowski, 1979). Rhoda Erdmann intended to change this when she published her practice book on tissue culture. She called it a “first attempt to spread the methodology to wider circles”. Students should learn the methods, to be able to use them “at free will” for later works. The methodology should become a “common good” for future biologists (Erdmann, 1922, Preface).

In the light of her role in the scientific community this attempt at establishing the field on a broader, common basis had a personal aspect for Erdmann, besides the propagation of a scientific discipline: knowledge and skills, not institutional backing or the number of laboratory assistants, should be the decisive factor in becoming a leader in the field. The foundation of her journal served the same purpose. She hoped to create a forum for in her view so far underrepresented works. She considered herself a democrat and linked it to her understanding of the organization of science. To Harrison, she wrote that she declined traditional structures like academies, because this contradicted her democratic thinking (S. Koch, 1985, 112). It seems consequential that she hoped to be able to catch up with the leaders in the field, although she had comparably lesser resources, by democratizing knowledge.

## Conclusion

A niche is usually perceived as a recess in a room. It can also mean a small section of a market or a space suiting “the character, capabilities, status, etc., of a person or thing”.^6^ This idea of the niche might help to explain why Rhoda Erdmann could become one of the first female professors at a time when it was hard for women to cope with the scientific system. Simultaneously, the analogy helps to explain why and how she tried to leave the sub-niche of culture media research to become a leader in the field of tissue culture research, more broadly conceived with its links to immunology, cancer research, and regeneration. Tissue culture media research was an academic niche from different points of view. In the 1910s and 1920s it was seen as a small section of regeneration research, structurally it was not yet institutionalized with its own academic departments or specialized journals, and in terms of spaces the research was conducted in various laboratories with basic facilities. Facing the topical narrowness and the few people involved, its umbrella, tissue culture research itself, had been an academic niche for many years before it could become an institutionalized discipline. Additionally, its status as a not-yet institutionalized research field with rather few centers beyond the major pillars, made it a possible niche for researchers who were about to start a career or who felt underprivileged in well-established fields of research after the First World War.

Rhoda Erdmann after her first years in Berlin found in the United States, in Harrison’s laboratory, the perfect fit between her microbiological working methods of raising (cell) cultures and a new thematic direction promising new insights into processes of regeneration, reproduction, and growth. Simultaneously, she found in Harrison a supporter who tried to help her academically and in private throughout her life. During her first imprisonment in the United States, Harrison tried to help her as much as he could and, when she was terrorized by the Nazi regime, Harrison travelled to Berlin to fight for her release (Niedobitek, Niedobitek, and Sauerteig, 2017, 198–209; Schneck, 2000, 178–180).

Populating a niche can be comfortable when it means that it comes along with less competition. But from the perspective of a scientist like Rhoda Erdmann, staying in the niche was unsatisfying. Science of the 20th century is to some extent, as Whitley has shown, a reputational system (Whitley, 1984). Recognition and self-constitution belong to the driving forces in scientific networks selecting and attributing attention and resources to ideas, experimental systems, and people (Fangerau, 2013). One of the most visible attributes of granted recognition is the association of a scientist with an institution representing his or her research field. Erdmann was striving for such a department when she returned to Germany, struggling to leave her small niche. When she noticed that she was not satisfied with the special niche of culturing media, she scaled up her interests and skills. Provided with the facilities of a cancer research institute, she created a new niche at the intersection of biology and medicine, which she called “experimental cell research with a special focus on tissue culture”. To compensate for her lack of resources, she tried to reduce the basic need for research funds by democratizing knowledge.

Rhoda Erdmann was never alone in her niche, but leaving it meant growing competition. She had her own (not independent) unit, but the first guest department of the Kaiser-Wilhelm Institute of Biology was granted to her competitor, Albert Fischer. Nevertheless, she succeeded in becoming highly visible in her field by founding an international journal, which was supported by all the stars of tissue culture. In terms of innovation theory she can be seen as an “early adopter” (Rogers, 1962) of tissue culturing, which made her a visible scholar from the second half of the 1920s until the late 1930s.

Did her legacy last? In his obituary Paul Caffier noted that he did not know which of her experimental works would be remembered in future, but he considered it beyond doubt that she was the person who retransferred the field of tissue culture research from the United States back to Germany, where it had been—according to his views—originally established by Curt Herbst and Wilhelm Roux (Caffier, 1936, 134f.). This Germano-centric statement might be read as being addressed to the National Socialist government ruling the country by that time. Michael Engel argued in 1994 that new research fields like tissue culturing offered possibilities for young and innovative researchers to find a niche, and that laboratories like Rhoda Erdmann’s offered the chance to try something new. At the same time this new research irritated the establishment. He sees the persecution of Erdmann, the closing of her department after her death and the cessation of her journal in 1944 as a reaction of the establishment, which found “ideological support” in the new NS government when it tried to get rid of unwanted scientists (Engel, 1994, 298–300).

After Erdmann’s death she and her role were indeed in danger of being eclipsed from history although the Swiss histologist Otto Bucher for example dedicated some lines and a photo to her in his historical overview of tissue culture published in the Ciba-Zeitschrift in 1940 (Bucher, 1940, 2530-2534). When the American Philipp R. White published a cell culture manual in 1954, he ignored Erdmann in his historical sketch of the discipline and—as if mirroring her—introduced the manual by stating that he wanted to “strip from the study of this subject its former atmosphere of mystery and complication”, in order to make it a common good (quoted from Witkowski, 1979, 280–281).

However, that is not the end of the story. Retrospectively, Erdmann was so successful in her science that at first a biographical memoir for Warren Lewis remembered her crucial role in establishing cell culturing. It stated that Erdmann had prepared the agar on which Margaret Reed grew the first *in vitro* mammalian cell culture (Corner, 1967, 332–333). She was subsequently mentioned in historical works on tissue culture before the first biographies remembering her appeared in German. Finally, she was honored by the naming of a park after her in Berlin in 2012, a street in Munich in 2015, and a building of the Humboldt University Berlin in 2016. On all these occasions, not only her role as a female professor was stressed but also her role as an academic pioneer, who tried to transform tissue culture from an elitist endeavor to an academic discipline. 70 years after her death, her vision of applying cell culturing for solving biological and medical problems linked to regeneration, development, and growth has become common knowledge, and she is seen as a former leader in an academic field which she helped to carve out from a niche.

## Footnotes

1 Flexner to Loeb 05.02.1917, Flexner papers, Archive American Philosophical Society; Erdmann to Loeb 31.01.1917, Loeb papers, Library of Congress, MSS30429, Box 4.

2 Erdmann to Schmidt-Ott 22.02.1926 and other correspondence, Archiv der Max-Planck-Gesellschaft, I 001 A 1449 07–0,011 to 0,026, here 0,025 and 0,026.

3 Klage des B. G. Teubner Verlags gegen Rhoda Erdmann wegen Nichterfüllung des Vertrages zur Verfassung eines “Lehrbuchs der Biologie für höhere Lehranstalten” und unberechtigter Forderungen. Sächsisches Staatsarchiv, 22,198 B. G. Teubner Verlag, Leipzig, Nr. 1,597.

4 Simon Flexner to Rhoda Erdmann 15.12.1924, Rockefeller Archive Center, Simon Flexner papers, Rockefeller Institute for Medical Research (RIMR), Subseries 1.2., Erdmann, Rhoda.

5 Rhoda Erdmann to Simon Flexner 8.11.1924 and 6.2.1925, Rockefeller Archive Center, Simon Flexner papers, Rockefeller Institute for Medical Research (RIMR), Subseries 1.2., Erdmann, Rhoda.

6 “niche, n. and adj.”. OED Online. September 2021. Oxford University Press. https://www.oed.com/view/Entry/126748 (Accessed September 29, 2021).
